# Targeting Proteolysis with Cyanogenic Glycoside Amygdalin Induces Apoptosis in Breast Cancer Cells

**DOI:** 10.3390/molecules27217591

**Published:** 2022-11-05

**Authors:** Valentina Cecarini, Salima Selmi, Massimiliano Cuccioloni, Chunmei Gong, Laura Bonfili, Yadong Zheng, Manuela Cortese, Mauro Angeletti, Soumaya Kilani, Anna Maria Eleuteri

**Affiliations:** 1 School of Biosciences and Veterinary Medicine, University of Camerino, Via Gentile III da Varano, 62032 Camerino, Italy; 2 Research Unit for Bioactive Natural Products and Biotechnology UR17ES49, Faculty of Dental Medicine of Monastir, University of Monastir, Avicenne Street, Monastir 5000, Tunisia; 3 Department of Pharmaceutical Sciences A, Faculty of Pharmacy of Monastir, University of Monastir, Avicenne Street, Monastir 5019, Tunisia; 4 CHiP Research Center, School of Pharmacy, University of Camerino, Via Madonna delle Carceri, 62032 Camerino, Italy

**Keywords:** amygdalin, proteasome, autophagy, apoptosis, cancer, apricot kernel extract

## Abstract

Background: Breast cancer is the most diagnosed cancer among women, and its incidence and mortality are rapidly growing worldwide. In this regard, plant-derived natural compounds have been shown to be effective as chemotherapeutic and preventative agents. Apricot kernels are a rich source of nutrients including proteins, lipids, fibers, and phenolic compounds and contain the aromatic cyanogenic glycoside amygdalin that has been shown to exert a cytotoxic effect on cancer cells by affecting the cell cycle, inducing apoptosis, and regulating the immune function. Methods: Here, we describe a previously unexplored proapoptotic mechanism of action of amygdalin in breast cancer (MCF7) cells that involves the modulation of intracellular proteolysis. For comparative purposes, the same investigations were also conducted upon cell treatment with two apricot kernel aqueous extracts from *Prunus armeniaca* L. Results: We observed that both the 20S and 26S proteasome activities were downregulated in the MCF7 cells upon 24 h treatments. Simultaneously, the autophagy cascade resulted in being impaired due to cathepsin B and L inhibition that also contributed to a reduction in cancer cell migration. The inhibition of these proteolytic systems finally promoted the activation of apoptotic events in the MCF7 cells. Conclusion: Collectively, our data unveil a novel mechanism of the anticancer activity of amygdalin, prompting further investigations for potential application in cancer preventative strategies.

## 1. Introduction

Cancer is a major public health burden in modern countries, and breast cancer is one of the most common cancers among women. Numerous factors, including age, obesity, family history, tobacco use, hormone therapy, etc., can increase the risk of breast cancer; at the same time, specific behavioral choices and nutritional practices can help to reduce the risk of this disorder, thus representing successful prevention strategies with no or negligible adverse effects [1]. In this regard, several plant-derived natural compounds have been shown to be effective as chemotherapeutic and preventative agents through the induction of different cellular mechanisms, among them programmed cell death [2,3,4]. In the last few years, significant progresses have been made in dissecting the biological activity of these compounds in both cellular and in vivo models. The apricot (*Prunus armeniaca* L.) belongs to the Rosaceae family and is mainly cultivated in the Mediterranean area. The fruit is a source of nutrients and phytochemicals with beneficial antioxidant, antimicrobial, anti-inflammatory, and antimutagenic properties [5,6,7]. In particular, apricot kernels (or seeds) are traditionally used to treat different kind of disorders such as skin disease, hemorrhages, spasms, inflammation, bronchitis, asthma, emphysema, and nausea. Kernels are a rich source of proteins, carbohydrates, vitamins, fibers, organic acids, phenols, volatile compounds, and lipids and contain the aromatic cyanogenic glycoside amygdalin that has been shown to induce apoptosis and cell cycle arrest in several human cell lines, including prostate, breast, lung, bladder, colon, and rectal cancer cells and keratinocytes [5,8,9,10,11,12]. In addition, amygdalin exerts an antitumor activity inhibiting metastatic spread through integrin regulation [13].

Interestingly, no data are currently available on the ability of amygdalin to modulate the activity of the proteolytic systems involved in cell proliferation, migration, and apoptotic events. The proteasome and autophagy are the two major degradative processes in cells. They are involved in the degradation of dysfunctional or damaged cellular components, including soluble proteins and whole organelles. They are intimately correlated, and numerous studies have demonstrated that dysfunctions in one degradative system may affect the other pathway [14,15]. The inhibition of these proteolytic processes can trigger cytotoxicity favoring the accumulation of aggregates, dysfunctional proteins, and organelles, finally inducing apoptotic events in cells [16,17,18].

In this study, amygdalin was investigated for its ability to modulate the activity of proteolytic enzymes such as the proteasome complex and lysosomal cathepsins B and L in normal MCF10A and human breast cancer MCF7A cells. In silico analyses were also performed to gain insight into the interactions between amygdalin and the active site of the tested enzymes. The activation of apoptotic events following proteolysis modulation was also dissected. Finally, considering that amygdalin is a signature compound of apricot kernels, we also explored the same pathways upon treatment with two different aqueous extracts (obtained by decoction and maceration) of apricot kernels.

## 2. Results

### 2.1. Evaluation of the Extracts Composition

**Figure 1:**
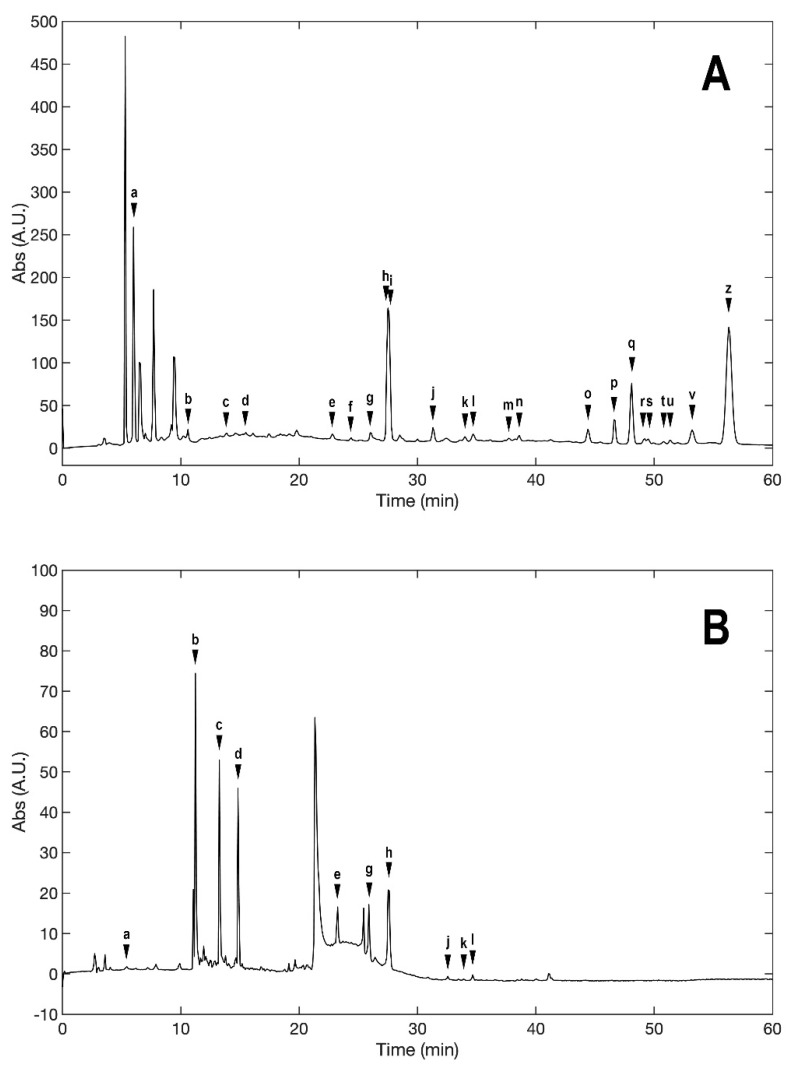
Phytochemical profile of the decoction (AKE (D), Panel (**A)**) and maceration (AKE (M), Panel (**B)**) extracts obtained upon chromatographic analysis. a, Shikimic acid; b, Gallic acid; c, Loganic acid; d, 5-caffeylquinic acid; e, Swertiamarin; f, Catechin hydrate; g, Delphinidin−3,5−diglucoside; h, Amygdalin; i, Sweroside; j, Chlorogenic acid; k, Vanillic acid; l, Caffeic acid; m, Epichatechin; n, Syringic acid; o, p-Coumaric acid; p, Ferulic acid; q, Naringin; r, Rutin hydrate; s, Hyperoside; t, Resveratrol; u, Amarogentin; v, Kaempferol−3−glucoside; z, Quercetin dihydrate.

LC-ESI-MS was used to evaluate the composition of the obtained extracts. Figure 1 (Panels A,B) shows the two chromatograms obtained upon analyses. It is evident that the extraction procedure influences the composition in terms of the bioactive compounds of the obtained products, with the decoction extract showing a higher monomeric polyphenol content with respect to the maceration extract. Table 1 shows the phytochemical content in the analyzed extracts, and the previous papers, if available, that observed the same compounds in kernel extracts are indicated. Shikimic acid, amygdalin, sweroside, naringin, and quercetin dihydrate were among the most abundant species present in the decoction extract, whereas gallic acid, loganic acid, and 5−caffeoylquinic acid were mainly detected in the maceration extract. The total phenolic content was determined in both extracts, and no significant variations were observed: 994 ± 54 mg GAE/100 g DW for the AKE (M) and 1134 ± 87 mg GAE/100 g DW for the AKE (D). Interestingly, extraction by decoction resulted in higher levels of amygdalin (Panel B).

### 2.2. Effect of Treatments on Cell Viability and Proliferation

**Figure 2:**
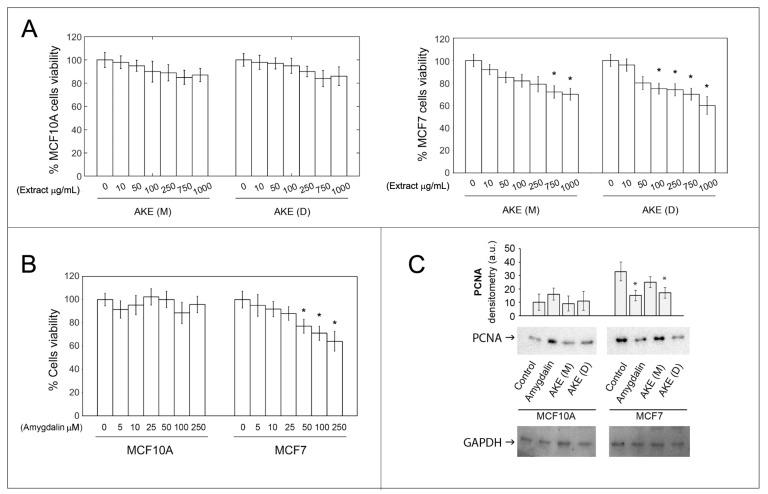
Effect of amygdalin and apricot kernel extracts on cell viability and PCNA levels. The MTT assay was carried out to measure cell viability in MCF10A and MCF7 cells. Cells were treated with increasing concentrations of maceration extract and decoction extract (Panel (**A)**) and amygdalin (Panel (**B)**) for 24 h. Results are expressed as percent toward untreated cells. Data points marked with an asterisk are statistically significant compared with the respective untreated control cells (* *p* < 0.05). (**C**) Representative Western blots for PCNA and relative densitometry is reported. GAPDH was used as control for equal protein loading. Data points marked with an asterisk are statistically significant compared with the respective untreated control cells (* *p* < 0.05).

Cellular viability was evaluated upon treatment with increasing concentrations of apricot kernel aqueous extracts (maceration AKE (M) and decoction AKE (D), 0–1 mg/mL) and amygdalin (0–250 µM) in MCF10A and MCF7 cells, using an MTT assay. Interestingly, both aqueous kernel extracts induced a dose-dependent cytotoxicity in the MCF7 cells, with the AKE (D) effects already evident at 100 µg/mL. Conversely, no signs of toxicity were detected in the MCF-10A cells. As for amygdalin, a decline in cell viability was detected in the MCF7 cells treated with concentrations ≥50 µM. Again, the MCF10A cells did not show any changes in cell viability (Figure 2, Panels A,B). The effects on MCF10A and MCF7 cell proliferation were further investigated through the detection of PCNA, an auxiliary factor for DNA polymerase σ involved in DNA replication, chromatin remodeling, sister chromatid cohesion, cell cycle control, and DNA repair [26]. The expression levels of PCNA significantly decreased in the MCF7 cells treated with amygdalin or with the decoction extract with respect to the untreated cells. No significant variations were detected in the normal cells upon treatments. The AKE (M) did not affect breast cancer cell proliferation (Figure 2, Panel C).

### 2.3. Effect of Treatments on Proteasome Functionality

**Figure 3:**
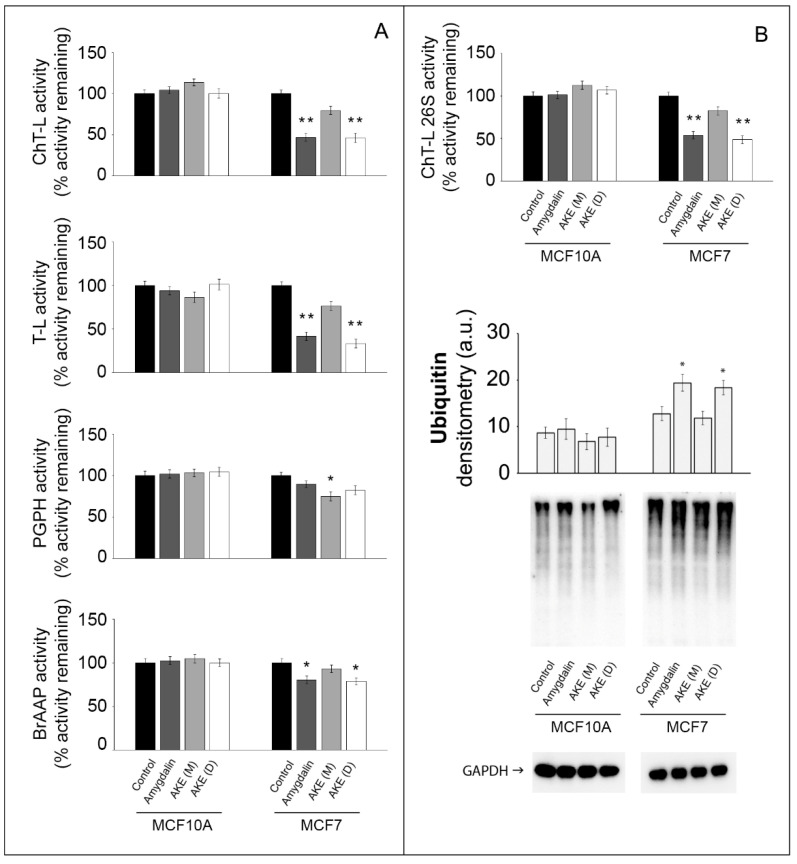
Effect of amygdalin and apricot kernel extracts on the 20S and 26S proteasome functionality. (**A**) 20S proteasome activities in normal and cancer cells following 24 h treatment with 50 µM amygdalin and 1 mg/mL apricot kernel extracts. Asterisks indicate significantly different values compared with respective untreated control cells (* *p* < 0.05, ** *p* < 0.01). Results are expressed as mean values and standard deviation and are obtained from five separate experiments. (**B**) 26S ChT-L proteasome activity in treated cells and a representative Western blot of ubiquitin-conjugates with the relative densitometry. GAPDH was used as equal loading control. * *p* < 0.05, ** *p* < 0.01 indicates significantly different values compared with respective untreated control cells. Results are expressed as mean values and standard deviation and are obtained from five separate experiments. Statistical analysis was performed with one-way ANOVA, followed by the Bonferroni test using SigmaStat 3.1 software.

The proteasome functionality was monitored by checking both the enzymatic activity and substrate accumulation in the treated cells. The chymotrypsin-like (ChT-L), trypsin-like (T-L), peptidylglutamylpeptide hydrolyzing (PGPH) and branched chain amino acid-preferring (BrAAP) proteasomal activities were measured in cell lysates using fluorogenic substrates (Figure 3).

The treatment with the two extracts and amygdalin did not affect the proteasome functionality in the normal MCF10A cells. Conversely, amygdalin and the AKE (D) induced an evident inhibition of the ChT-L and T-L activities of the 20S proteasome in the MCF7 cells. A minor but still significant effect was observed on the BrAAP activity. The PGPH component of the 20S proteasome in the MCF7 cells showed a peculiar trend, being only affected by the AKE (M) treatment. We also analyzed the functionality of the 26S proteasome measuring the ChT-L activity. This complex is constituted by the 20S core and two additional 19S subunits and needs ATP and ubiquitinated substrates for final degradation. No effect was observed in the MCF10A cells, whereas a significantly decreased activity of the 26S proteasome in the cancer cells was observed upon treatment with amygdalin and AKE (D) (Figure 3A). To gain insight into the UPS modulation, we detected the levels of ubiquitinated proteins. Ub levels notably increased in the MCF7 cells treated with amygdalin or with the AKE (D) compared with untreated cells. Such an increase correlates with the declined activity obtained upon the enzymatic assays. As expected, no significant variations were detected in the normal cells after treatments (Figure 3B).

### 2.4. Effects of Treatments on Apoptosis

**Figure 4:**
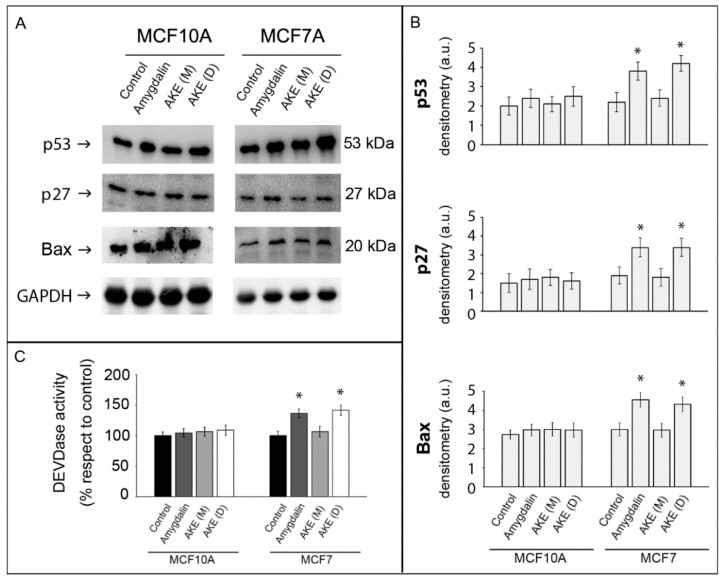
Effect of amygdalin and apricot kernel extracts on apoptosis markers. Representative Western blot of p53, p27, and Bax (Panel (**A)**) and relative densitometry (Panel (**B)**). GAPDH was used as equal loading control. Results are expressed as mean values and standard deviation and are obtained from five separate experiments. * *p* < 0.05 indicates significantly different values compared with respective untreated control cells. (**C**) DEVDase activity was measured in normal and cancer cells following 24 h treatment with 50 µM amygdalin and 1 mg/mL apricot kernel extracts. Asterisks indicate significantly different values compared with respective untreated control cells (* *p* < 0.05). Results are expressed as mean values and standard deviation and are obtained from five separate experiments. Statistical analysis was performed with one-way ANOVA, followed by the Bonferroni test using SigmaStat 3.1 software.

Proteasome inhibition can exert anticancer effects by inducing the accumulation of proteins involved in cell proliferation and cell death mechanisms [27]. Therefore, we investigated whether the treatments activate apoptotic pathways in MCF7 cells by evaluating the amount of apoptosis-related proteins such as p53, p27, and Bax that are also known targets of proteasome-mediated degradation [28]. In line with the proteasome inhibition, the three proteins were upregulated upon MCF7 cell exposure to amygdalin and AKE (D), suggesting the activation of apoptotic mechanisms in these cells, whereas no effect was detected in the normal MCF10A cells (Figure 4, Panels A,B). The DEVDase activity assay measures the activity of caspase-3-like proteases, such as caspase-3 and caspase-7. No changes in the DEVD substrate degradation were detected in the normal MCF10A cells, whereas the increased fluorescent values in the caspase-3-deficient MCF-7 cells [29] treated with amygdalin and the AKE (D) suggested that caspase-7 was the enzyme involved (Figure 4, Panel C).

### 2.5. Effect of Treatments on Autophagy Functionality

**Figure 5:**
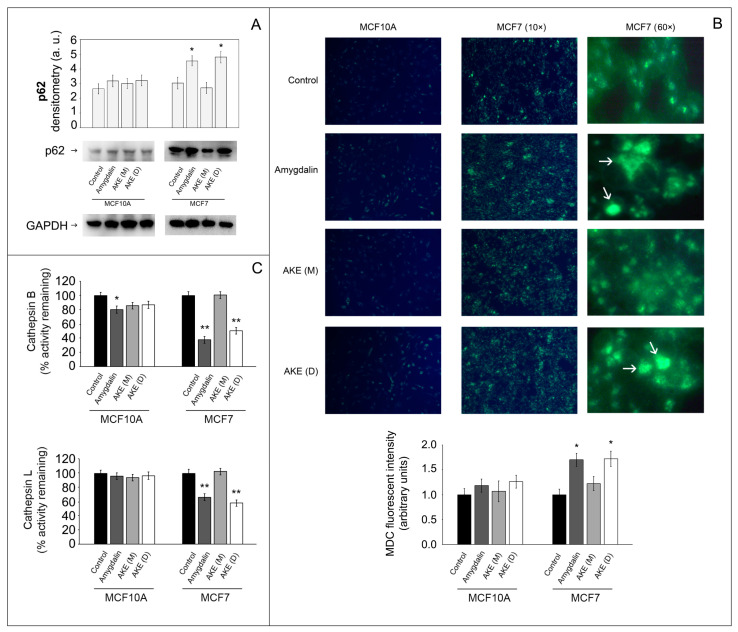
Effect of amygdalin and apricot kernel extracts on autophagy. (**A**) A representative Western blot of p62 protein and relative densitometry. GAPDH was used as equal loading control. Results are expressed as mean values and standard deviation and are obtained from five separate experiments. Statistical analysis was performed with one-way ANOVA, followed by the Bonferroni test using SigmaStat 3.1 software. * *p* < 0.05 indicates significantly different values compared with respective untreated control cells. (**B**) MDC staining of MCF10A and MCF7 treated cells. Cells were treated with amygdalin and kernel extracts and then exposed to the autofluorescent dye MDC to detect autophagic vacuoles. 10× and 60× magnifications are shown for MC7 cells. * *p* < 0.05 indicates significantly different values compared with respective untreated control cells. (**C**) Cathepsin B and cathepsin L activity measured in control and treated cells. Activity was measured using fluorogenic peptides as substrates as described in the Materials and Methods section. Data are indicated as percentage vs. untreated control cells (* *p* < 0.05, ** *p* < 0.01).

Next, we evaluated the effects of apricot kernel extracts on markers of the autophagic pathway, the other major proteolytic system in cells. First, we monitored the expression of the sequestosome1 (p62/SQSTM1, hereafter p62), a multidomain protein that acts as a signaling hub for multiple pathways, including protein turnover via the UPS and autophagy [14]. p62 is degraded in autophagolysosomes, and thus its levels inversely correlate with the autophagic activity. As shown in Figure 5A, a significant accumulation of p62 was observed in the cancer cells treated with amygdalin and with the AKE (D), suggesting an impairment of the autophagy process. No significant differences were observed in the p62 levels in the MCF10A cells upon treatments.

To further understand whether autophagy is affected by the treatments, we evaluated the formation of autophagic vacuoles using monodansylcadaverine (MDC) staining. MDC is an autofluorescent molecule that accumulates in mature autophagic vacuoles, including autophagolysosomes. The results show that treatments with amygdalin or with AKE (D) induce an increase in the fluorescent intensity and number of autophagic vacuoles in cancer cells (Figure 5B). Finally, we measured the activity of cathepsin B and cathepsin L, two major lysosomal cysteine proteases essential in controlling lysosomal function and involved in tumorigenesis and metastasis [30]. The activity of both enzymes was significantly decreased in the MCF7 cells treated with amygdalin or AKE (D) compared to the untreated cells (** *p* < 0.01). No difference was observed in the MCF10A cells upon treatment; only a minor decrease in cathepsin B activity upon amygdalin exposure. These data suggest that the occurrence of impaired autophagy in the treated MCF7 cells that promoted the accumulation of p62 and vacuoles is likely due to the inhibited cathepsin B and L activities.

### 2.6. Molecular Docking Analysis

**Figure 6:**
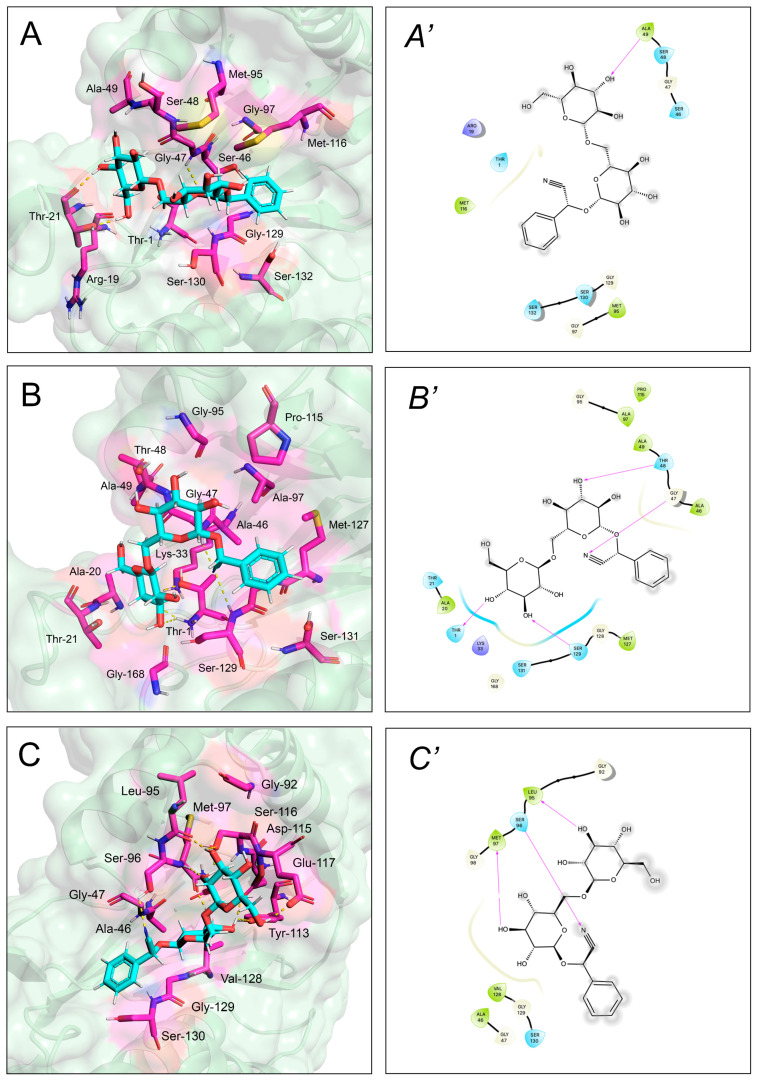
Computed binding modes of amygdalin to β1, β2, and β5 subunits of human constitutive 20S proteasome (pdb ID: 6rgq). Panels (**A**–**C**) report the comparative visualization of 3D models of amygdalin and the residues close to the active sites of β1, β2, and β5 subunits, respectively, that are directly involved in the formation of the enzyme-inhibitor complexes (displayed as light blue and pink sticks, respectively). H-bonds are indicated as yellow dashed solid lines. Panels (**A’**–**C’**): comparative 2D visualization of amygdalin and the residues close to the active site of β1, β2, and β5 subunits, respectively, that are directly involved in the formation of H-bonds (purple arrows), polar (light blue ribbon), and VdW interactions (green ribbon). Atoms/groups exposed to solvent are indicated with grey circles.

A structural rationale for the observed effects toward proteasome and cathepsin proteolytic activities was provided by in silico analyses of the interactions between amygdalin and the active site of the enzymes of interest. Amygdalin showed comparable moderate binding affinities for the proteasome catalytic subunits, which resulted from a balanced contribution between vdW and electrostatic energy terms to the stabilization of the complexes (the K_D_ values and energy contributions are summarized in Table 2). Structurally, amygdalin was predicted to establish interactions with amino acid residues close to the proteasome catalytic sites, with a resulting binding geometry being likely to prevent substrate access to the catalytic Thr-1 (Figure 6).

**Figure 7:**
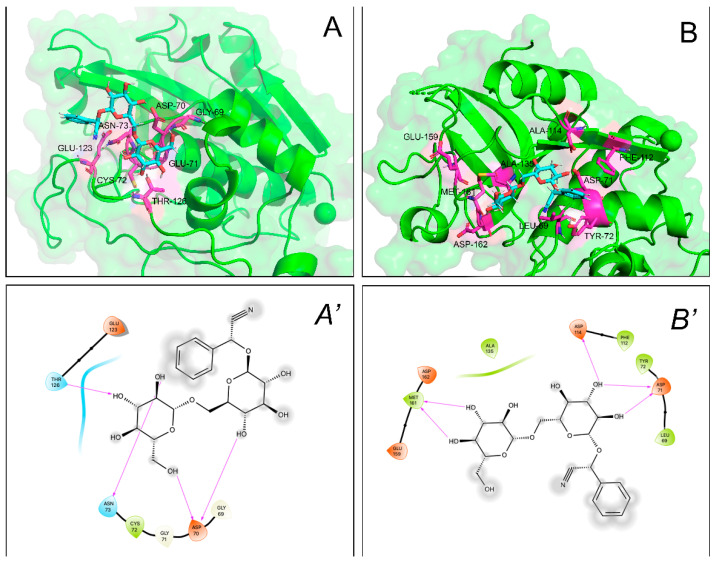
Computed binding modes of amygdalin to cathepsin B (PDB ID: 1csb) and L (PDB ID: 3hha). Panels (**A**,**B**) report the comparative visualization of 3D models of amygdalin and the residues close to the active site of cathepsin B and L, respectively, that are directly involved in the formation of the enzyme-inhibitor complexes (displayed as light blue and pink sticks, respectively). H-bonds are indicated as yellow dashed solid lines. Panels (**A’**,**B’**) report the comparative 2D visualization of amygdalin and the residues close to the active site of cathepsin B and L, respectively, that are directly involved in the formation of H-bonds (purple arrows), polar (light blue ribbon), and VdW interactions (green ribbon). Atoms/groups exposed to solvent are indicated with grey circles.

Irrespective of a similar hindering effect on the substrate accessibility to the catalytic Cys-29 (Figure 7), amygdalin showed approx. 5–10 lower affinity for cathepsins compared to proteasome, with the contribution of the electrostatic energy prominent in establishing complex stability (Table 3).

### 2.7. Inhibition of Cell Migration

**Figure 8:**
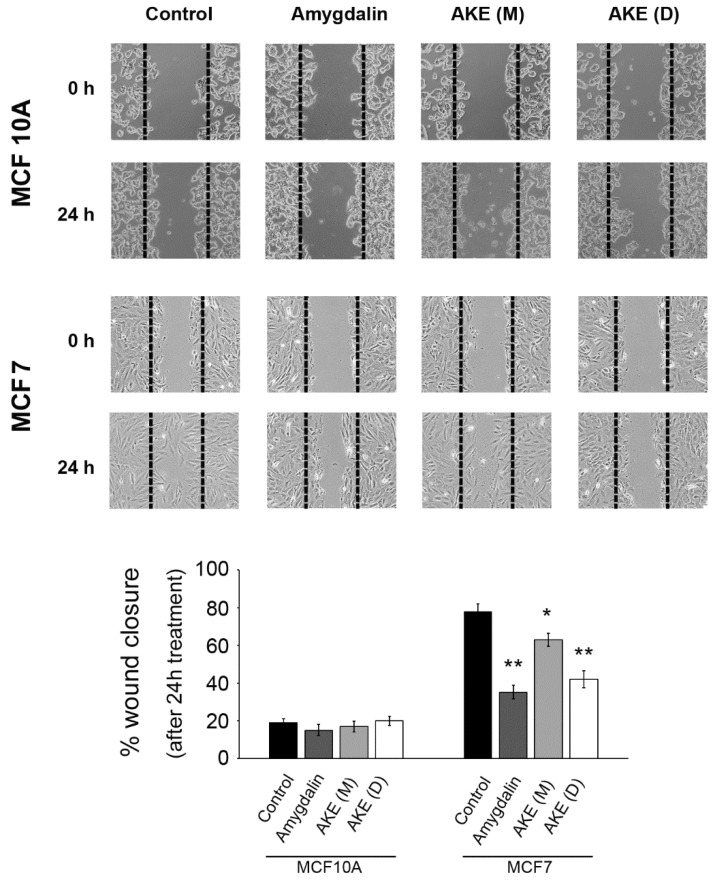
Effect of treatments on cell migration. Confluent monolayers were scratched, and wound closure was monitored using a microscope equipped with a camera after 24 h exposure to amygdalin or extracts. Each experiment was repeated at least five times. Results were expressed as percentage wound closure (* *p* < 0.05 and ** *p* < 0.01 compared to control).

Considering the data obtained measuring the cathepsin activity and the role of these enzymes in cancer progression, we performed a scratch motility assay to monitor whether the apricot kernel extract also affects cancer cell migration. The wound healing was evaluated 24 h after scratching (Figure 8). Again, amygdalin and the decoction extract significantly retarded wound closure in the MCF7 cancer cells after 24 h. The untreated control was almost completely closed. The maceration extract also delayed the closure but with a lower efficacy. No significant effect on MCF10A cell motility was observed.

## 3. Discussion

The regular consumption of adequate amounts of fruit and vegetables may reduce the incidence of cancer, and this evidence raised interest in the therapeutic use of these foods as natural sources of anticancer agents. This possibility has triggered the attention of researchers, and currently a huge body of evidence supports the anticancer action of naturally occurring compounds [31,32]. Being a rich source of health beneficial nutrients (carbohydrates, vitamins, minerals, fibers, etc.) and phytochemicals (polyphenolics, carotenoids, and glycosides), apricot has become a product of great interest, particularly for its functional properties. In particular, apricot kernels are a good reservoir of secondary metabolites and of amygdalin, which has been previously studied and characterized for its anticancer effects [9,33].

In this study, we investigated the effect of amygdalin and of two aqueous apricot kernel extracts on the functionality of proteolytic systems (in detail, proteasome and autophagy) and the implications on apoptotic events in MCF7 human breast carcinoma cells and in the normal counterpart (MCF10A cells). Generally, we found that the two different extraction procedures influence the phytochemical composition of the extracts, whereas no major differences were obtained with the TPC. Conversely, a different content in amygdalin was detected that was higher in the AKE (D). The MCF7 cells treated with aqueous extracts showed comparable reductions in cell viability (30% and 40% reduction with AKE (D) and AKE (M), respectively) likely due to the similar composition in terms of the total phenolic content. No changes in viability were detected in the MCF10A cells. PCNA expression, an index of cell proliferation, was affected upon treatment with amygdalin and AKE (D) but not with the AKE (M).

The effects of amygdalin and both extracts were then tested on proteasome functionality, measuring the activity of both the 20S catalytic core and the 26S proteasome. Both proteolytic complexes were inhibited in the cancer cells treated with the decoctum, whereas no variation in the activities was detectable in the same cells treated with the AKE (M) extract. Interestingly, the extent of proteasomal inhibition was comparable to that of isolated amygdalin. The inhibition of the proteasomal complex was confirmed by accumulation of its substrates p53, p27, and Bax that are proteins involved in the cell cycle and apoptotic process, suggesting the possible activation of this pathway of cell death [34]. The upregulation of the DEVDase activity, that indicates the activation of the effector caspase-7 in caspase-3-deficient MCF7 cells, unequivocally confirms the occurrence of apoptotic events. Although this is not the first study to indicate the proapoptotic activity of amygdalin [9,11], our data show the ability of this compound to exert its effect through the modulation of proteasome activity and its catalytic components.

We then investigated the effects of the treatments on autophagy, the other main catabolic intracellular mechanism that intensively cooperates with the proteasome in regulating several processes. Numerous studies previously demonstrated that the activity of the two systems is mutually regulated [14,35]. Autophagy is a finely regulated multistep process that mainly contributes to the maintenance of cellular homeostasis through the degradation and recycling of cytoplasmic components and organelles in the lysosomes, thus showing a physiological cytoprotective role [36,37]. Cathepsin B and L are involved in the autophagic process and responsible for the final degradation of the lysosomal content [38,39]. Herein, we demonstrate that amygdalin and the apricot kernel decoction extract significantly inhibit the functionality of both cathepsins in cancer cells, favoring the impairment of the autophagic cascade as indicated by accumulation of p62 protein and autophagic vacuoles. The occurrence of dysfunctional autophagic flux, together with the impaired proteasomal system, finally favors the activation of apoptotic events with the involvement of caspase-7. The connection between the two processes, with autophagy inhibition favoring apoptosis, was previously explored by other studies. In line with our findings, the natural flavonoid fisetin exhibited a selective anticancer activity in caspase-3-deficient MCF7 cells through the induction of apoptosis and inhibition of autophagy [40]. Liu and colleagues demonstrated that IMB-6G was able to induce autophagy-dependent apoptosis through an autophagosomal–cathepsin axis in pancreatic cancer cells [41]. Interestingly, there are several autophagy inhibitors that, by blocking this process, sensitize cancer cells to therapy, enhancing the cytotoxic effects of the associated chemotherapeutic agent [42]. Among them, the antimalarial drug chloroquine acts as an autophagy inhibitor, which triggers apoptosis in different cellular models [43,44]. The ability of the extract to target lysosomal cathepsins is particularly remarkable considering the overexpression of cathepsins in several types of cancer. Bengsch and colleagues demonstrated in mice models of breast cancer that the overexpression of cathepsin B increased tumor cell invasion favoring proteolytic extracellular matrix degradation [45]. Similarly, higher levels of cathepsin L were reported in kidney and testicular tumors and in most cancers of the breast, ovary, colon, adrenal, bladder, prostate, and thyroid [46,47]. Thus, cysteine cathepsins are an attractive target in cancer therapy mainly for their role in the degradation of extracellular matrix that facilitates tumor cells growth, invasion, and metastasis. The finding that our treatments also retarded wound closure in the MCF7 cancer cells further confirms the role of cathepsin inhibition in blocking cell migration and invasion.

The findings obtained in the present work demonstrate that the different phytochemical composition of the two aqueous extracts makes AKE (D) the most effective treatment. In particular, the presence of shikimic acid, sweroside, naringin, and quercetin dehydrate together with the higher amount of the cyanogenic glycoside amygdalin in AKE (D) favored its ability to affect the investigated proteolytic processes and to promote the activation of apoptotic events in the MCF7 cells, in line with previous works from our and other laboratories demonstrating that the flavonoids naringin and quercetin significantly inhibit the growth of various cell cancer cell lines and target the proteasomal system [44,48,49,50].

## 4. Conclusions

Growing studies recently reported on the ability of natural extracts to inhibit tumor cell growth and metastasis and induce apoptosis in cancer cells with different specificity and molecular targets and with a final effect, depending on the combination of their active components. The results shown here provide novel mechanisms for amygdalin and apricot kernel extract-induced apoptotic processes in breast cancer cells that involve the inhibition of cellular proteolytic pathways. These properties make this extract worthy of further in-depth studies for therapeutic development and potential applications in cancer preventative strategies.

## 5. Materials and Methods

### 5.1. Chemicals and Reagents

Amygdalin (analytical standard) was purchased from Extrasynthese (Genay Cedex, France). Gallic acid, catechin, chlorogenic acid, caffeic acid, epicatechin, rutin, iperoside, resveratrol, and quercetin (analytical standards) were purchased from Sigma-Aldrich S.r.L. (Milan, Italy). Cell lines were from the American Type Culture Collection (ATCC, Rockville, MD, USA). All the media, reagents, and plastics for cell cultures were purchased from Euroclone (Milan, Italy). 3-(4,5-Dimethyl-2-thiazolyl)-2,5-diphenyl-2H-tetrazolium bromide (MTT) used in cytotoxicity assays was from Merck Spa (Milan, Italy). The substrates Suc-Leu-Leu-Val-Tyr-AMC, Z-Leu-Ser-Thr-Arg-AMC, and Z-Leu-Leu-Glu-AMC for assaying the chymotrypsin-like (ChT-L), trypsin-like (T-L), and peptidyl glutamyl-peptide hydrolyzing (PGPH) activities of the proteasomal complex were purchased from Sigma-Aldrich S.r.L. (Milano, Italy). The substrate Z-Gly-Pro-Ala-Leu-Ala-MCA to test the branched chain amino acids preferring (BrAAP) activity was obtained from Biomatik (Cambridge, Ontario, Canada). Aminopeptidase N (EC 3.4.11.2) for the coupled assay utilized to detect BrAAP activity was purified from pig kidney, as reported elsewhere [51]. Membranes for Western blot analyses were purchased from Millipore (Milano, Italy). Proteins immobilized on films were detected with the enhanced chemiluminescence (ECL) system (Amersham Pharmacia Biotech, Milan, Italy).

### 5.2. Extraction Procedures and Determination of Polyphenols by HPLC

Apricots (cultivar ‘Chechi Khit El Oued’) were collected in Monastir, a city in eastern Tunisia. Apricot kernels were separated, dried, crushed to powder and extracted according to two extraction protocols. Specifically, the kernel powder was either extracted with distilled water for 72 h under mild and constant stirring at room temperature (extraction by maceration) or by boiling water for 20 min at 98 °C (extraction by decoction). Resulting extraction solutions were independently filtered using 20 µm filters, freeze-dried, and stored in sealed vials until use. Polyphenols were characterized through chromatographic analyses carried out on an AKTA HPLC System (GE Healthcare, Milan, Italy), equipped with a UV-vis detector. Separation was conducted on a Phenomenex Luna C18 column (5 µm particle size, 250 × 4.6 mm; column volume (CV): 4.116 mL). Elution was performed with a mobile phase consisting of a mixture of 3% acetic acid in water (eluent A) and acetic acid, CH_3_CN, and water (3:25:72 *v*/*v*, eluent B). The samples were eluted according to the following segmented gradient: 0–80% B in 8 CV, 80–90% in 1 CV; injection volume 10 mL, detection performed at 280 nm, flow rate: 1 mL/min. Identification of compounds was achieved by comparison of their retention times with those of standard solutions and/or by LC-ESI-MS in negative ionization mode. The injection volume was 5 μL. The temperature of the column was 30 °C, and the temperature of the drying gas in the ionization source was 350 °C. The gas flow was 12 L/min, the nebulizer pressure was 60 psi, and the capillary voltage was 4000 V (negative and positive). Detection was performed by ESI-MS in the ‘multiple reaction monitoring’ (MRM) mode. To enhance the sensitivity, the acquisition time was divided into three periods. The most abundant product ions were used for the identification of each analyte.

### 5.3. Determination of Total Phenolic Content

Total phenolic content (TPC) was estimated by the Folin–Ciocalteu assay [52]. Each dried extract (50 mg) was dissolved in deionized water (50 mL) and sonicated for 2 min. A 1 mL aliquot of this solution was added to 79 mL of water and 5 mL of Folin–Ciocalteu reagent and allowed to react for 1 min. Then, 15 mL of 200 g/L NaHCO_3_ was added, and the resulting mixture was incubated for 2 h. The absorbance was read at 760 nm against a blank sample. A gallic acid solution was used as standard, and data were expressed as mg of gallic acid equivalents (GAE) per 100 g dry weight (DW).

### 5.4. Cell Lines

MCF7 cells were grown in minimum essential medium (MEM) supplemented with 10% fetal bovine serum (FBS), 1% penicillin/streptomycin, and 1% sodium pyruvate. MCF10A cells were cultured in DMEM/Ham’s F-12 (1:1), supplemented with 20 ng/mL epidermal growth factor (EGF), 10 µg/mL insulin, 500 ng/mL hydrocortisone, 5% equine serum, and 1% penicillin/streptomycin. Cells were grown at 37 °C in a 5% CO_2_-containing atmosphere.

### 5.5. Cell Treatment and Cell Viability Assay

For cell treatment, amygdalin and apricot kernel extracts were dissolved in growth medium, vortexed, and sonicated at 50 °C for 30 min to ensure complete solubilization. Control cells were included in each time point. After 24 h treatment, the medium was removed, and cells were washed and harvested in 4 mL of PBS and centrifuged at 1600× *g* for 5 min. The pellet was resuspended in lysis buffer (20 mM Tris, pH 7.4, 250 mM sucrose, 1 mM EDTA, and 5 mM β-mercaptoethanol) and passed through a 29-gauge needle at least ten times. Lysates were centrifuged at 12,000× *g* for 15 min, and the supernatants were stored at −80 °C until use. Protein concentration was determined by the method of Bradford, using bovine serum albumin (BSA) as standard [53]. Cell viability was evaluated with the 3-(4,5-dimethylthiazol-2-yl)-2,5-diphenyltetrazolium bromide assay (MTT) [54]. Upon 24 h treatment with increasing concentrations of amygdalin (0–250 µM) or the extracts (0–1 mg/mL), cells were washed in PBS, pH 7.5, and then MTT (final concentration 0.5 mg/mL) was added to the culture medium without FBS and incubated for 2 h at 37 °C. The medium was then removed and replaced with 100 μL of DMSO. The optical density was measured at 550 nm in a microtiter plate reader. At least six cultures were utilized for each time point.

### 5.6. Docking Analyses

Individual molecular models of the complexes between amygdalin and the catalytic subunits of human proteasomes were obtained according to flexible ligand-receptor docking using Autodock 4 [55], as previously reported [56]. Briefly, source files of both ligand and receptors were submitted to AutoDockTools and hydrogen atoms were added. Then, 3D model of amygdalin (PubChem ID: 656516) was docked onto the crystallographic structures of human constitutive proteasome (PDB ID: 6rgq [57]) within a 20 × 20 × 20 Å grid box (grid spacing: 0.375 Å) placed around the catalytic β1, β2, and β5 and extending 10 Å in each direction from the catalytic Thr-1 residue. The amygdalin–cathepsin B/cathepsin L models were analogously obtained, and the ligand–enzyme interactions were evaluated in a grid box placed around the catalytic site of 3D structure of human cathepsin B (PDB ID: 1csb [58]) and L (PDB ID: 3hha [59]) spanning 10 Å in each direction around the catalytic Cys-29. All residues in a radius of 5 Å around catalytic Thr-1 and Cys-29 (for proteasome and cathepsins, respectively) were selected as flexible residues. Default settings were used throughout. Resulting models were analyzed using Maestro (Schrödinger, LLC, New York, NY, USA, 2014-2) and rendered with PyMOL (the PyMOL Molecular Graphics System, Version 2.4 Schrödinger, LLC).

### 5.7. Proteasome Activity

The effects on the proteasome system were evaluated through fluorometric assays, as previously reported [60], using the following synthetic substrates: Leu-Leu-Val-Tyr-AMC for ChT-L, Leu-Ser-Thr-Arg-AMC for T-L, Leu-Leu-Glu-AMC for PGPH, and Gly-Pro-Ala-Leu-Ala-AMC for BrAAP, whose test was performed with the addition of the aminopeptidase-N (AP-N). The incubation mixture contained 1 μg of cell lysate, the appropriate substrate, and 50 mM Tris/HCl pH 8.0, up to a final volume of 100 μL. Incubation was performed at 37 °C, and after 60 min, the fluorescence of the hydrolyzed 7-amino-4-methyl-coumarin (AMC) was recorded (AMC, λ_exc_ = 365 nm, λ_em_ = 449 nm) on a SpectraMax Gemini XPS microplate reader. The 26S proteasome ChT-L activity was tested using Suc-Leu-Leu-Val-Tyr-AMC as substrate and 50 mM Tris/HCl pH 8.0 buffer containing 10 mM MgCl_2_, 1 mM dithiothreitol, and 2 mM ATP. The effective 20S proteasome contribution to short peptide cleavage was evaluated with control experiments performed using specific proteasome inhibitors, Z-Gly-Pro-Phe-Leu-CHO and lactacystin (5 μM in the reaction mixture). The fluorescence values of lysates were subtracted from the values of control assays in the presence of the two inhibitors.

### 5.8. Cathepsin B and Cathepsin L Activity

Cathepsin B and L proteolytic activities were measured using the fluorogenic peptides Z-Arg-Arg-AMC and Z-Phe-Arg-AFC, respectively, at a final concentration of 5 μM. The mixture for cathepsin B, containing 1 μg of cell lysate, was preincubated in 100 mM phosphate buffer pH 6.0, 1 mM EDTA, and 2 mM dithiothreitol for 5 min at 30 °C. Upon the addition of the substrate, the mixture was incubated for 15 min at 30 °C. The mixture for cathepsin L, containing 1 μg of proteins, was incubated in 100 mM sodium acetate buffer pH 5.5, 1 mM EDTA, and 2 mM dithiothreitol for 5 min at 30 °C and, upon the addition of the substrate, the mixture was incubated for 15 min at 30 °C. The fluorescent signal released by the hydrolyzed 7-amino-4-methyl-coumarin (AMC, λ_exc_  =  365 nm, λ_em_  =  449 nm) and 7-amino-4-trifluoromethylcoumarin (AFC, λ_exc_  =  397 nm, λ_em_  =  500 nm) was detected on a SpectraMax Gemini XPS microplate reader.

### 5.9. DEVDase Activity

DEVDase activity was measured in cell lysates (20 µg of total proteins in the mixture) using the Ac-Asp-Glu-Val-Asp-AMC substrate in 50 mM Tris-HCl, 50 mM NaCl, 5 mM CaCl_2_, 1 mM EDTA, 0.1% CHAPS, and 5 mM β-mercaptoethanol, pH 7.5. Incubation was carried out at 37 °C for 60 min, and the release of AMC was monitored on a SpectraMax Gemini XPS microplate reader (AMC: λ_exc_ = 365 nm, λ_em_ = 449 nm).

### 5.10. Western Blotting Analysis

Proteins were resolved on SDS–PAGE and electroblotted onto PVDF membranes. Membranes with transferred proteins were incubated with the primary monoclonal antibody and successively with the specific peroxidase-conjugated secondary antibody. Monoclonal antibodies were obtained from Santa Cruz Biotechnology, Inc. (Heidelberg, Germany). The immunoblot detection was performed with ECL Western blotting detection reagents using a ChemiDoc MP system. Each gel was loaded with molecular weight markers from 12 to 225 kDa (GE Healthcare). Glyceraldehyde-3-phosphate dehydrogenase (GAPDH) was utilized as a control for equal protein loading: membranes were stripped and reprobed with anti-GAPDH monoclonal antibody (Santa Cruz Biotechnology, Heidelberg, Germany). Stripping buffer contained 200 mM glycine, 0.1% SDS, and 1% Tween 20. Immunoblot images were quantified using ImageJ 1.52a software (NIH, Bethesda, MD, USA). 

### 5.11. Monodansylcadaverine Assay

Upon treatments, the formation of autophagic vacuoles was monitored with the monodansylcadaverine assay (MDC, Sigma-Aldrich S.r.L. Milan, Italy). In detail, 1 µM MDC was added to cell medium. After 10 min incubation at 37 °C, cells were washed three times with phosphate buffered solution (PBS) and immediately analyzed with a fluorescence microscope Olympus IX71 (Olympus, Tokyo, Japan). Fluorescence intensity was evaluated by ImageJ 1.52p software.

### 5.12. Cell Migration Analysis

A scratch motility assay was performed to monitor the effect of treatments on cancer cell migration, as previously described [3]. Briefly, cells were seeded at 6 × 10^5^ cells per mL in a 6-well microtiter plate and grown to confluence. The monolayers were scratched with a 10 μL tip, and after treatments, cells migrating into the cell-depleted zone (the scratched area) were counted at 5 randomly selected fields. Cell motility was expressed as percentage wound closure with respect to untreated cells. Cell-covered areas and percentage of wound closure were calculated at 24 h using ImageJ 1.52a software (NIH, Bethesda, MD, USA).

### 5.13. Statistical Analysis

Data are expressed as mean values ± S.D. Statistical analysis was performed with one- way ANOVA, followed by the Bonferroni post hoc test using SigmaStat Version 3.1 software (SPSS, Chicago, IL, USA), and *p* < 0.05 was considered statistically significant.

## Figures and Tables

**Table 1:** Compounds detected in the decoction (AKE(D) and maceration (AKE(M) extract obtained upon chromatographic analysis. Retention time, parent and product ion *m/z* values, and peak areas for both extracts are indicated. n.d.: not detected. * compounds identified with the internal standard.

Peak ID	Compound	Chemical Formula	Chemical Structure	Retention Time (min)	Precursor Ion (*m/z*)	Product Ion (*m/z*)	AKE-D Peak Area (mAU*min)	AKE-M Peak Area (mAU*min)	References
a	Shikimic acid	C_7_H_10_O_5_	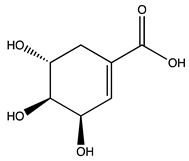	6.11	173	173	51.96	>2	
b	Gallic acid *	C_7_H_6_O_5_	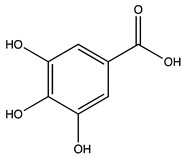	11.02	169	125	>2	25.33	[19,20]
c	Loganic acid	C_16_H_24_O_10_	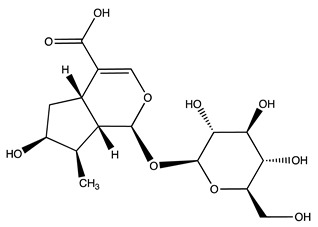	13.13	375	213	>2	16.54	
d	5−caffeylquinic acid	C_16_H_18_O_9_	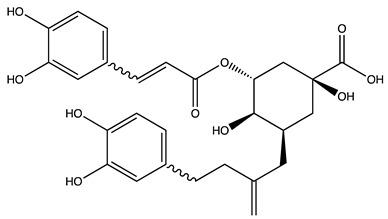	15.52	353	191	>2	13.77	[21]
e	Swertiamarin	C_16_H_22_O_10_	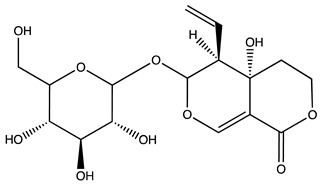	23.02	419	179	3.16	4.43	
f	Catechin hydrate *	C_15_H_16_O_7_	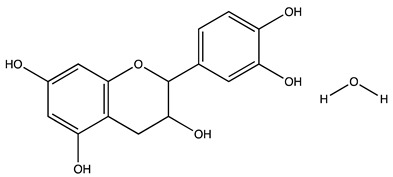	24.06	289	245	>2	n.d.	[19,21]
g	Delphinidin-3,5-diglucoside	C_27_H_30_O_17_	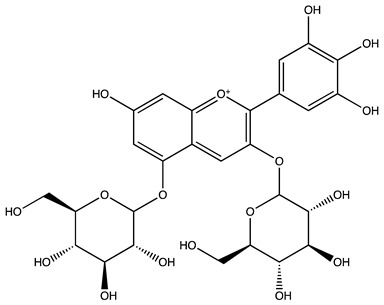	25.84	463	300	3.42	6.77	
h	Amygdalin *	C_20_H_27_NO_11_	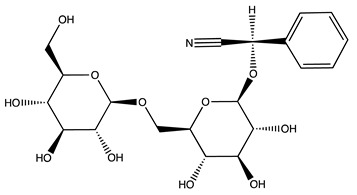	27.52	456	323	48.57	8.22	[21,22,23]
i	Sweroside	C_16_H_22_O_9_	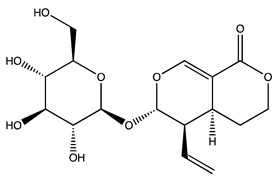	27.70	403	125	18.86	n.d.	
j	Chlorogenic acid *	C_16_H_18_O_9_	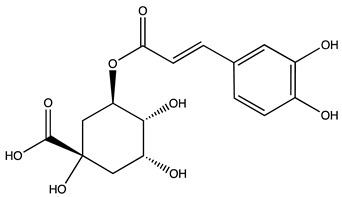	31.53	353	191	6.13	>2	[20]
k	Vanillic acid	C_8_H_8_O_4_	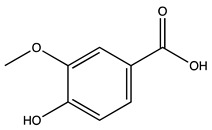	33.81	167	108	>2	>2	[24]
l	Caffeic acid *	C_9_H_8_O_4_	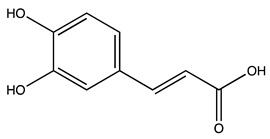	34.52	179	135	4.03	>2	[19,20]
m	Epicatechin *	C_15_H_14_O_6_	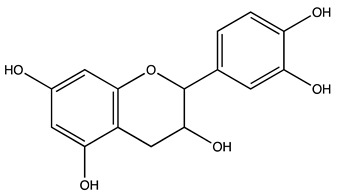	37.62	289	245	>2	n.d.	[20,21]
n	Syringic acid	C_9_H_10_O_5_	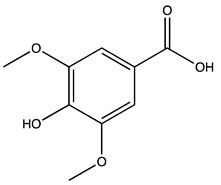	38.48	197	182	2.64	n.d.	[19]
o	p−Coumaric acid	C_9_H_8_O_3_	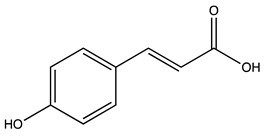	43.82	163	119	6.47	n.d.	[19]
p	Ferulic acid	C_10_H_10_O_4_	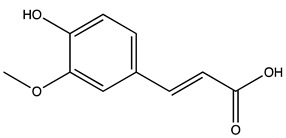	46.51	193	134	9.70	n.d.	[19]
q	Naringin	C_27_H_32_O_14_	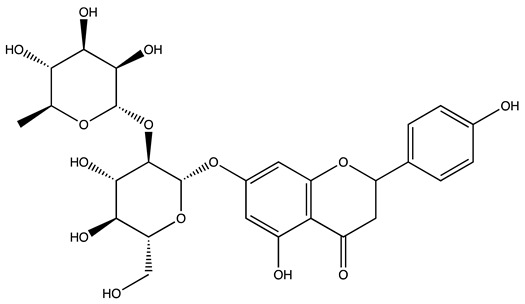	47.72	579	271	26.61	n.d.	
r	Rutin hydrate *	C_27_H_32_O_17_	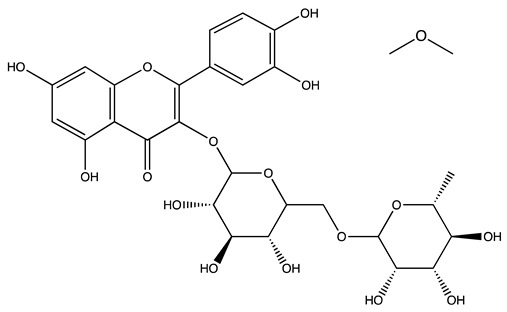	48.59	609	300	>2	n.d.	[19,20]
s	Hyperoside *	C_21_H_20_O_12_	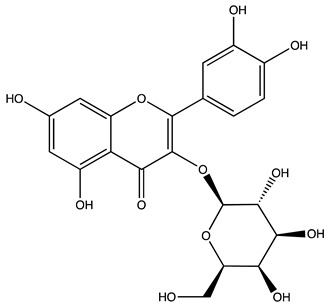	49.11	463	300	>2	n.d.	
t	Resveratrol *	C_14_H_12_O_3_	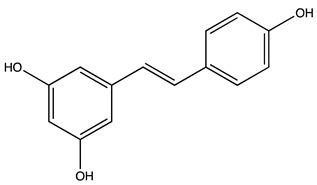	51.13	227	185	>2	n.d.	[19]
u	Amarogentin	C_29_H_30_O_13_	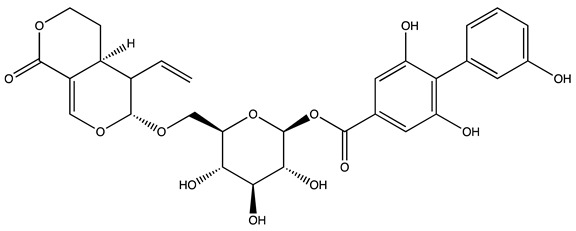	51.71	585	227	>2	n.d.	[25]
v	Kaempferol−3−glucoside	C_21_H_20_O_11_	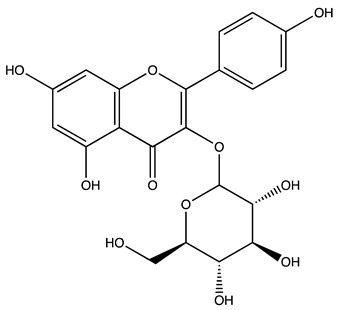	53.62	447	284	8.17	n.d.	[19]
z	Quercetin dihydrate *	C_15_H_14_O_9_	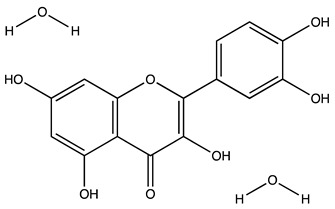	56.68	301	151	100.90	n.d.	[19]

**Table 2:** Predicted affinities and energy contribution values for the complexes formed between human constitutive 20S proteasome catalytic subunits and amygdalin. I. Energy: Internal energy term consists of torsional energy, repulsion at short distances, van der Waals and electrostatic contributions. T. Energy: total energy term consists of Internal energy + electrostatic and van der Waals contributions to the intermolecular interactions.

Name	ΔG (kcal mol^−1^)	K_D_ (μM)	T. Energy (kcal mol^−1^)	I. Energy (kcal mol^−1^)	vdW Energy (kcal mol^−1^)	Electrostatic Energy (kcal mol^−1^)
Beta1	−7.981	1.41	59.523	−37.852	−22.906	−14.946
Beta2	−7.891	1.64	62.034	−34.950	−20.883	−14.067
Beta5	−7.659	2.43	52.792	−50.518	−14.204	−36.314

**Table 3:** Predicted affinities and energy contribution values for the complexes formed between the human cathepsins and amygdalin. I. Energy: Internal energy term consists of torsional energy, repulsion at short distances, van der Waals and electrostatic contributions. T. Energy: total energy term consists of Internal energy + electrostatic and van der Waals contributions to the intermolecular interactions.

Name	ΔG (kcal mol^−1^)	K_D_ (μM)	T. Energy (kcal mol^−1^)	I. Energy (kcal mol^−1^)	vdW Energy (kcal mol^−1^)	Electrostatic Energy (kcal mol^−1^)
Cathepsin B	−6.706	12.1	71.224	−32.369	−6.705	−25.664
Cathepsin L	−7.113	6.10	69.395	−37.456	−7.838	−29.618

## Data Availability

Data are contained within the article.

## Sample Availability

Samples of the compounds are not available from the authors.
